# How Do Economic Growth, Urbanization, and Industrialization Affect Fine Particulate Matter Concentrations? An Assessment in Liaoning Province, China

**DOI:** 10.3390/ijerph17155441

**Published:** 2020-07-28

**Authors:** Tuo Shi, Yuanman Hu, Miao Liu, Chunlin Li, Chuyi Zhang, Chong Liu

**Affiliations:** 1 CAS Key Laboratory of Forest Ecology and Management, Institute of Applied Ecology, Chinese Academy of Sciences, No. 72, Wenhua Road, Shenyang 110016, China; tuoshi0411@163.com (T.S.); huym@iae.ac.cn (Y.H.); trullyzhang@163.com (C.Z.); liuchong@iae.ac.cn (C.L.); 2 College of Resources and Environment, University of Chinese Academy of Sciences, No. 19, Yuquan Road, Beijing 100049, China

**Keywords:** air pollution, fine particulate matter, economic growth, urbanization, industrialization, Granger causality test

## Abstract

With China’s rapid development, urban air pollution problems occur frequently. As one of the principal components of haze, fine particulate matter (PM_2.5_) has potential negative health effects, causing widespread concern. However, the causal interactions and dynamic relationships between socioeconomic factors and ambient air pollution are still unclear, especially in specific regions. As an important industrial base in Northeast China, Liaoning Province is a representative mode of social and economic development. Panel data including PM_2.5_ concentration and three socio-economic indicators of Liaoning Province from 2000 to 2015 were built. The data were first-difference stationary and the variables were cointegrated. The Granger causality test was used as the main method to test the causality. In the results, in terms of the causal interactions, economic activities, industrialization and urbanization processes all showed positive long-term impacts on changes of PM_2.5_ concentration. Economic growth and industrialization also significantly affected the variations in PM_2.5_ concentration in the short term. In terms of the contributions, industrialization contributed the most to the variations of PM_2.5_ concentration in the sixteen years, followed by economic growth. Though Liaoning Province, an industry-oriented region, has shown characteristics of economic and industrial transformation, policy makers still need to explore more targeted policies to address the regional air pollution issue.

## 1. Introduction

Since implementing the reform and opening-up policy in 1978, China has been experiencing a rapid process of social and economic development, attracting worldwide attention [1,2]. With the development of China’s urbanization process, the population influx into cities, the consumption of resources and the transformation of the economic structure have caused a variety of social and environmental impacts [3,4]. Among them, air pollution is particularly prominent because it is closely associated with negative health effects [5,6].

In recent years, one of the primary pollutants most affecting China has been fine particulate matter (PM_2.5_). PM_2.5_ refers to small particles or droplets in the air less than 2.5 microns in aerodynamic diameter [7,8]. PM_2.5_ easily binds to toxic and harmful substances due to its small size, long atmospheric residence time and extensive atmospheric transportation and seriously affects human health [9,10]. PM_2.5_ exposure in 2015 was estimated to result in 8.9 million deaths globally, among which 28% occurred in China [11]. To cope with severe and persistent PM_2.5_ pollution and to meet pollutant concentration targets [12,13], it is urgent and necessary to explore the influence of human factors on PM_2.5_ [14,15,16]. Hao and Liu [17] used a spatial lag model and spatial error model to investigate the socioeconomic influencing factors of urban PM_2.5_ concentration in China. The results showed that the number of vehicles and the secondary industry had significantly positive effects on PM_2.5_ concentration in cities. Wang et al. [18] found a positive correlation between PM_2.5_ concentrations and urban area, and population and proportion of secondary industry, and determined the existence of an inverted U-shaped relationship between economic growth and PM_2.5_ concentration. Existing studies have confirmed the contributions of socioeconomic factors to PM_2.5_ pollution in China [13,19,20]; however, the dynamic relationships and causal interactions between them are still not well understood, especially in specific regions. The Granger causality test determines the causal relationships between variables based on the chronological order in which the events occurred [21]. The method has been widely used in the empirical analysis of the relationships between energy, environment, economic and social development, etc. [22,23,24]. As an important administrative unit of a country, “province” usually provides unified and periodic suggestions to the cities under its jurisdiction, but relevant studies at this scale were few. Understanding the principal environmental issues in each stage of development holds great significance for the formulation and implementation of pollution policy, and also for the improvement of public health in China with PM_2.5_ as the primary pollutant.

In this paper, the panel data from 2000 to 2015 in Liaoning Province that combine a satellite derived PM_2.5_ concentration data set and socioeconomic data were established. The panel Granger causality test was used as the main method to quantitatively test the causality among economic growth, urbanization, industrialization and PM_2.5_ concentration. This study provides an idea for the formulation of regional periodic pollution control objectives which is significant to regional pollution control.

## 2. Materials and Methods

### 2.1. Study Area

**Figure 1:**
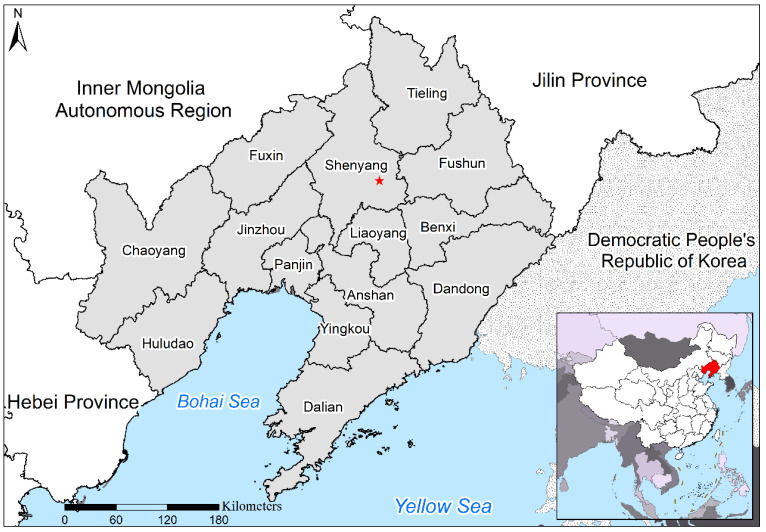
Location and cities in the study area.

Liaoning Province is located in Northeast China, covering an area of 148,000 km^2^, including 14 prefecture-level cities (Figure 1). The population is 43.82 million, including 29.52 million urban residents. Liaoning Province is a region in Northeast China where cities characterized by heavy industry are concentrated.

In Liaoning Province, the secondary industry accounted for 48.12% of the total GDP in 2015, with the province ranking 5th among the 31 provinces in China. In April 2015, TomTom, the Dutch traffic navigation service provider, released a global traffic congestion ranking, and Shenyang, the capital of Liaoning Province, ranked 29th [25]. According to data from the China National Environmental Monitoring Centre, 11 out of 14 cities in Liaoning Province experienced severe air pollution in November 2015. Therefore, there is an urgent need to study the relationships and interactions between socioeconomic factors and ambient pollution in Liaoning Province.

### 2.2. Data

**Figure 2:**
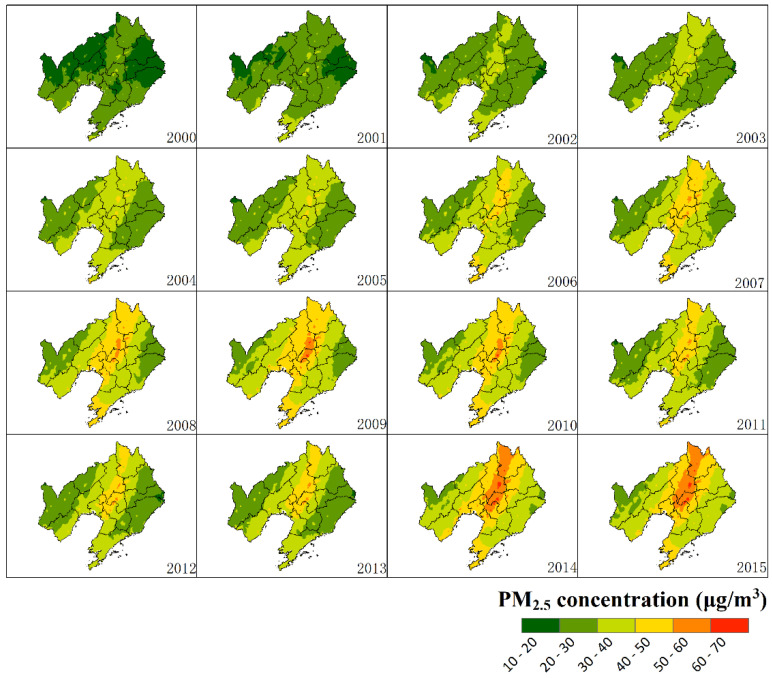
Spatial distribution of surface PM_2.5_ concentrations in Liaoning province from 2000 to 2015.

The annual PM_2.5_ concentrations from 2000 to 2015 in the panel data were extracted from the global PM_2.5_ concentration with a spatial resolution of 0.01° (http://fizz.phys.dal.ca/~atmos/martin/?page_id=140#V4.CH.02) [13,26,27,28]. The global PM_2.5_ concentration data set was implemented by the atmospheric chemistry driven model GEOS-Chem. The algorithm in the model combines the aerosol optical depth obtained from multi-sensor products with the data from surface monitoring stations [13,29,30]. The correlation coefficient of the estimated and regulatory monitored PM_2.5_ concentration was 0.81 [28]. To avoid uncertainty in the subsequent analysis caused by abnormal or missing values in the data, the three-year average was used as an annual average. The average PM_2.5_ concentrations from 2000 to 2015 in 14 prefecture-level cities were extracted and calculated by city boundaries (Figure 2).

Referring to relevant studies, we selected GDP per capita (GDPPC), the proportion of urban impervious surface area (UIS) and the value added by industry as a percentage of GDP (IND) to represent the economic growth, urbanization and industrialization of each city, respectively [22]. The panel data on the economic growth and industrialization of the fourteen prefecture-level cities in Liaoning Province from 2000 to 2015 were collected from the China City Statistical Yearbook. Because China has cancelled the agricultural and non-agricultural household registration system since 2014, to avoid abnormal fluctuation of time series data, the proportion of urban artificial impervious surface area rather than the traditional proportion of urban population was used to express the urbanization level of each city [31]. The spatial resolutions of 30 m urban artificial impervious area data were obtained from Fine Resolution Observation and Monitoring of Global Land Cover (FROM-GLC, http://data.ess.tsinghua.edu.cn/urbanChina.html) [31,32]. The GDPPC data were converted to constant prices, and all data were logarithmically transformed to stabilize the time series data and reduce the heteroscedasticity when performing empirical tests (*ln*PM_2.5_, *ln*GDPPC, *ln*UIS and *ln*IND). 

### 2.3. Methodology

The procedure for estimating the causal relationships between PM_2.5_ and the above socioeconomic factors using the panel data from 2000 to 2015 included five steps: the unit root test, panel cointegration test, panel fully modified least squares (FMOLS) regression, Granger causality test, variance decomposition and impulse response. The details are as follows:

A unit root test checks whether the unit root exists and if a time series variable is non-stationary [33]. If there is a unit root in the time series variable, it will lead to a pseudo-regression in subsequent regression analysis [34]. The null hypothesis is defined as the existence of a unit root, and the variables are non-stationary. In this study, the methods of Levin, Lin and Chu (LLC) and Im, Pesaran and Shin (IPS) were used for testing.

A panel cointegration test is used to test whether there is a long-term stable equilibrium relationship between variables. In this study, the Pedroni method was used to test the cointegration relationship between the socioeconomic variables and PM_2.5_ concentrations [16].

The panel FMOLS regression designed by Phillips [35] is utilized to provide the optimal estimations of cointegrating regressions [36]. This method modifies least squares to account for the autocorrelation effects and the endogeneity in the regressors due to the existence of a cointegration relationship [35,37]. In this study, the panel FMOLS regression was used to explore the trends and directions of *ln*GDPPC, *ln*UIS and *ln*IND in *ln*PM_2.5_ in the long term. The relationship between variables was expressed by the following equation, Equation (1):

*ln*PM_2.5*it*_ = *α*+ *β_1_ln*GDPPC*_it_* + *β_2_ln*UIS*_it_* + *β_3_ln*IND*_it_* + *ε_it_* (1)

where *i* and *t* represent the city and the time indexes in the panel, as shown by subscripts *i* (*i* = 1, …, 14) and *t* (*t* = 1, …, 16), respectively. α is the intercept; *β*s are partial coefficients of *ln*GDPPC, *ln*UIS and *ln*IND; and *ε*s refer to errors.

The panel vector error correction model (VECM) was used to investigate the direction and Granger causal relationships between the variables in the panel in the short or long term. In this study, short-term causality represented weak Granger causality because the dependent variable only responds to the short-term shocks of the stochastic environment (a stochastic environment refers to the agent’s actions and does not uniquely determine the outcome), whereas long-term causality referred to the independent variable’s response to the deviation from long-term equilibrium [22,38]. Generally, short-term causality affected 1–2 periods, while long-term causality represented the casual relationship of the whole period from 2000 to 2015 [22]. The short-term Granger causality depended on the χ^2^-Wald statistics of the coefficient significances of the lagged terms of the explanatory variables [38]. The long-term Granger causality was determined by the error correction term (ECT) significance. If the variables are cointegrated, then the coefficients of the ECTs are expected to be at least one or all negative and significantly different from zero [22].

Variance decomposition explains the amount of information each endogenous variable contributes to the other variables in the autoregressions. The impulse response function indicates the effects of a shock to one innovation on current and future values of the endogenous variables [38,39]. The Cholesky decomposition technique was used in the VECM to determine the contribution of one variable on another and estimate how each variable responds to the changes in the other variables [22].

The above methods were realized in the software EViews 8.0 (IHS Global Inc., Englewood, CA, USA), and relevant statistical principles were followed according to the user guide [40,41].

## 3. Results

### 3.1. Data Description

The PM_2.5_ concentrations data used in the study were extracted from the global data set provided by Van Donkelaar, Martin, Brauer and Boys [28]. In his study, sample points outside North America and Europe had precision with a correlation coefficient of 0.81 and a slope of 0.68. However, given the regional differences, the precision of the data involved in the study in Liaoning Province was yet to be verified.

**Figure 3:**
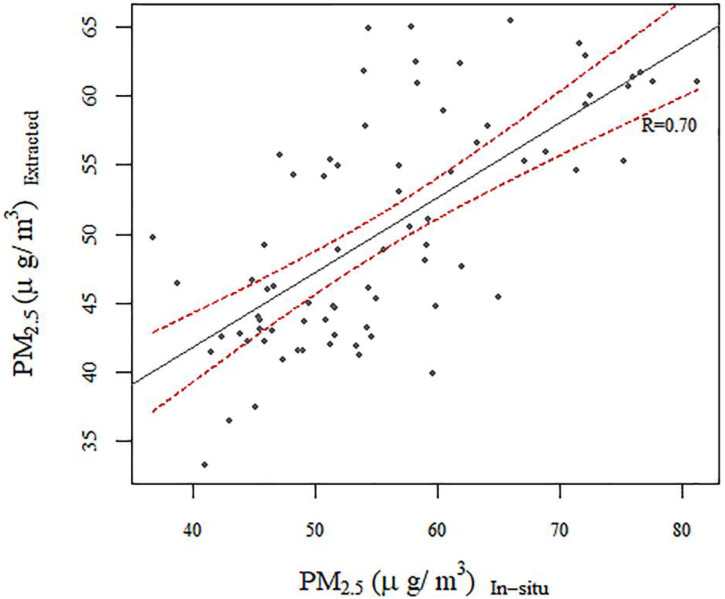
Scatter plot of regulatory stations that monitored PM_2.5_ concentrations and remote-sensed PM_2.5_ concentrations. Dashed red lines represent a 95% confidence interval of the fitting line.

Only in 2013 did the monitoring of particulate matter begin in various cities of China. Among them, cities in Liaoning Province started to have stable and continuous monitoring data from May 2014. Therefore, we selected the 76 regulatory stations that monitored PM_2.5_ values in 2015 for verification, and the correlation coefficient was 0.7 (Figure 3). Additionally, Peng, Chen, Lü, Liu and Wu [29] compared 45 sample points values from published studies and the corresponding remote-sensing values in China, with 78.7% correlation. Therefore, it is reasonable to believe that the data can reflect the variation of PM_2.5_ concentrations in the region and can be used for the following analysis.

The PM_2.5_ concentration, GDPPC, UIS and IND of fourteen cities in Liaoning Province from 2000 to 2015 were selected; the descriptive statistics are summarized in Table 1.

**Figure 4:**
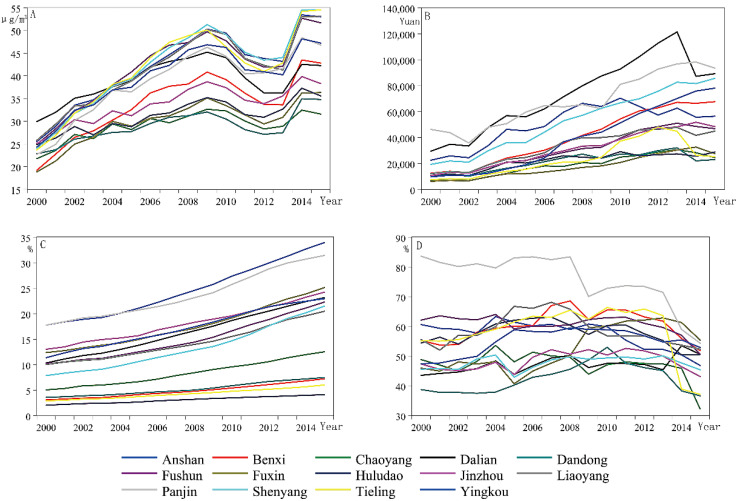
Data of PM_2.5_ concentrations (**A**), GDP per capita (GDPPC) (**B**), proportion of urban impervious surface area (UIS) (**C**) and the value added by industry as a percentage of GDP (IND) (**D**) of fourteen cities in the panel that changed over the time series from 2000 to 2015.3.2. Panel Unit Root Test Results.

Since 2000, PM_2.5_ concentration has been on the rise in fourteen cities in Liaoning Province, except for a temporary decrease from 2009 to 2012, and after 2014, the concentration also weakened (Figure 4). Increasing trends also occurred in the GDPPC and UIS, but after 2013, the economic growth of most cities slowed down or even declined. The changes of UIS in fourteen cities were basically stable, and most cities showed faster increasing trends after 2009. Regarding IND, the proportions in all cities decreased after 2012, indicating a characteristic of industrial transformation, or that the contribution of industrialization to economic growth has declined.

### 3.2. Panel Unit Root Test Results

The results (Table 2) showed that not all the variables in the panel were stationary at the levels; however, the four variables were basically stationary at the first difference. Therefore, we can reject the null hypothesis and assume the panel variables were stationary at the first difference.

### 3.3. Panel Cointegration Test Results

The results (Table 3) showed that six statistics could significantly reject the null hypothesis that there was no cointegration relationship; that is, a long-term stable cointegration relationship between PM_2.5_ concentration and explanatory variables existed in our panel data.

### 3.4. Panel Fully Modified Least Squares (FMOLS) Regression Results

The results are shown in Table 4, indicating that economic growth, urbanization and industrialization all had long-term positive effects on changes in PM_2.5_ concentrations in the sixteen years.

### 3.5. Panel Granger Causality Test Results

**Figure 5:**
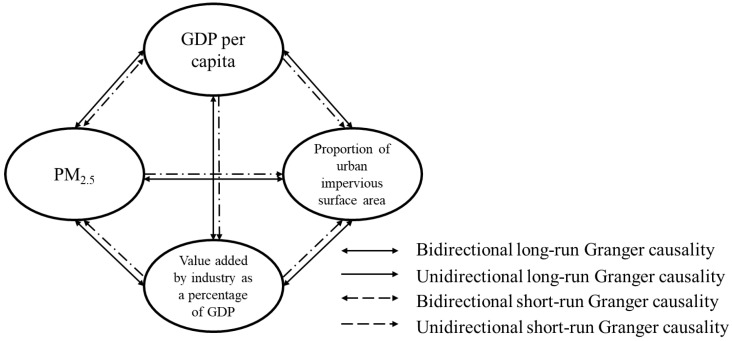
Diagram of the causal relationships between PM_2.5_ concentrations, GDP per capita (GDPPC), the proportion of urban impervious surface area (UIS) and the value added by industry as a percentage of GDP (IND).

Table 5 showed that all the coefficients of ECT (-1) of variables were significant; that is, bidirectional and long-term causal relationships existed between both variables in the panel. According to the χ^2^-Wald statistics, bidirectional short-term causal relationships between PM_2.5_ concentrations and GDPPC were found in the structure. In addition, one-way short-term causalities were found from IND to PM_2.5_ concentrations and UIS, from GDPPC to IND and UIS and from PM_2.5_ concentrations to UIS. A more visual and clearer figure is shown (Figure 5) based on the above results.

In the panel, all socioeconomic variables caused the variations of PM_2.5_ concentrations in Liaoning Province, especially economic growth, which not only influenced changes in pollutant concentrations in the long and short term but also affected the changes in industrialization and urbanization in the long and short term. Additionally, industrialization directly caused changes in pollutant concentrations in the long and short run and caused variations in urbanization in the short and long term (Figure 5).

### 3.6. Variance Decomposition and Impulse Response Analysis Results

The results of the variance decomposition analysis in Table 6 compared the contribution of each variable to the changes in PM_2.5_ concentration. In the panel, the variances of PM_2.5_ concentration were mostly explained by its own standard shock (80.95%) in the 16-year period, while the contributions from the GDPPC, IND and UIS to the PM_2.5_ concentration were 9.20%, 9.56% and 0.29%, respectively.

**Figure 6:**
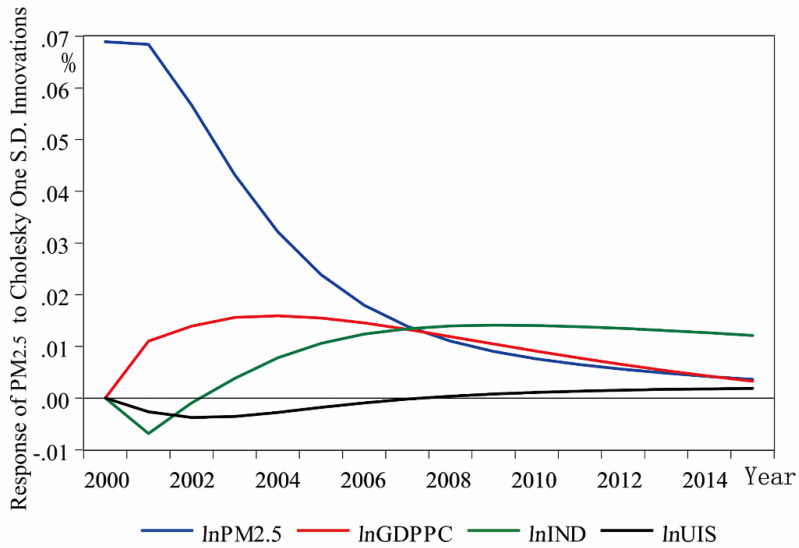
Results of the impulse response of *ln*PM_2.5_ to Cholesky one S.D. innovations of the variables.

The impulse responses result presented in Figure 6 showed that the responses of the PM_2.5_ concentration to itself decreased because of shocks from decreasing UIS and IND in the first two years. Then, from the fifth year, the response of the PM_2.5_ concentration continued to decrease because of shocks from decreasing GDPPC and decreasing IND in the latest seven years. 

## 4. Discussion

### 4.1. The Analysis of Relationships between PM_2.5_ and Socio-Economic Development in Liaoning Province 

Studies have shown that changes in fine particulate pollution concentrations in China are influenced by natural factors and human activities [42,43]. Therefore, to explore the impacts of urban socioeconomic factors on PM_2.5_ concentrations, we selected three indicators: urban GDP per capita, the proportion of urban impervious surface area and the value added by industry as a percentage of GDP, representing economic growth, urbanization and industrialization, respectively, which were assumed to be the most significant socioeconomic factors affecting PM_2.5_ concentrations in China. In our results, all selected socioeconomic variables were long-term causalities of the changes of PM_2.5_ concentrations, and economic growth and industrialization also significantly affected the variations in PM_2.5_ concentrations in the short term. The variance decomposition results showed that industrialization was the determinate factor affecting PM_2.5_ concentration variations in Liaoning Province, which was basically the same with the results found by Li, Fang, Wang and Sun [22], but only five cities in Liaoning Province were included in their industry-oriented panel, and the study period and indicators were different from ours. This further confirmed the attribute of Liaoning Province as a socio-economic mode of industry-oriented development.

Liaoning Province is an area in Northeast China where cities characterized by heavy industry are concentrated. Equipment manufacturing, the coal industry, the metallurgy industry and commodity production are the strengths of Liaoning Province [44]. For a long time period, heavy industry had been the main driving force of economic growth of most cities in Liaoning Province, promoting the rapid urbanization process. The concentrating population and developing economies would also motivate the urban industrial activities [45]. However, with the popularization and development of technology, the pressure of market competition increases. As a result, the supply of products in Liaoning Province far exceeds the market demand, and the problem of overcapacity is becoming increasingly serious [44]. Following the third scientific and technological revolution, the new science and technology industry, represented by electronics, computers, biological engineering, etc., seriously impacted traditional industries, resulting in a decline in the proportion of primary and secondary industries and leading to the rise of emerging industries such as the internet industry. However, in Liaoning Province, the tertiary industry only accounted for 38.7% of GDP in 2013, 5.8% less than the national average [46]. In 2015, the tertiary industry as a percentage of GDP rose to 46.06%, with major growth, basically equal to the national average. The slowdown in economic growth (Figure 4B) and the increase in the proportion of the tertiary industry indicated that adhering to the transformation of economic structure and industrial structure is a policy with both opportunities and difficulties. However, in recent years, the PM_2.5_ concentration has declined (Figure 4A), further proving the validity of industrial structure transformation.

### 4.2. The Analysis of Environmental Kuznets Curve (EKC) 

Although industrialization contributed the most to the PM_2.5_ concentration changes in the sixteen years in Liaoning Province, the contribution of economic growth dominated a longer period (Table 6). Moreover, some relationships between the economic growth and PM_2.5_ concentration changes were also noteworthy, such as the feedback effects in the Granger causality test (Figure 5) and fluctuations in the impulse response of shocks (Figure 6). Therefore, we constructed a regression model based on the Environmental Kuznets Curve (EKC) theory to study the relationship between economic growth and PM_2.5_ pollution. Grossman and Krueger [47] found that an inverted U-shaped relationship existed between economic growth and environmental pollution [48]. With a low level of economic development in a country or region, the degree of environmental pollution is relatively low, and with an improved economic level, the degree of environmental pollution intensifies. However, when economic development reaches a certain level, that is to say, reaches an “inflection point”, environmental quality gradually improves thenceforth with the increase in income.

**Figure 7:**
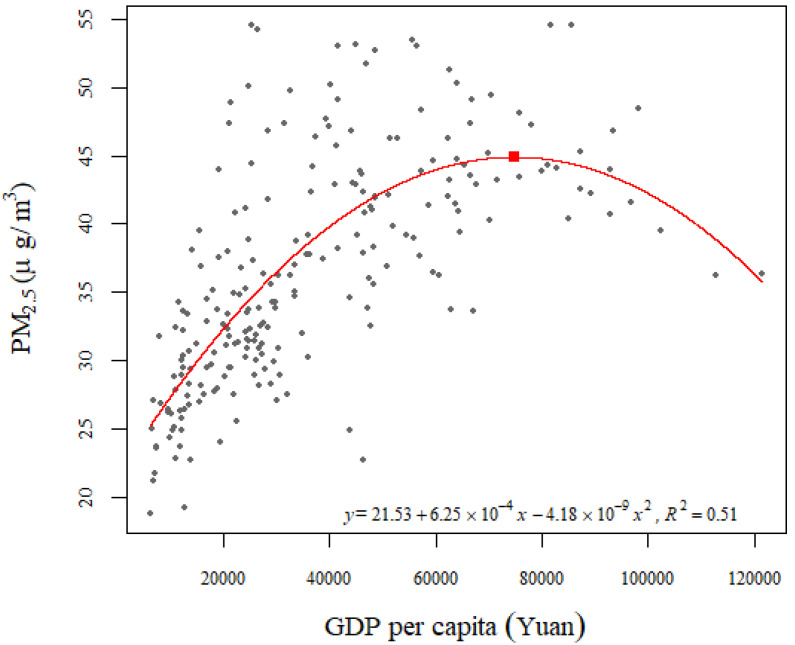
Scatter plot and Environmental Kuznets Curve (EKC) fitting line between PM_2.5_ concentrations and GDP per capita.

Our result of the EKC regression between GDPPC and PM_2.5_ is shown in Figure 7. According to the model equation, when the GDPPC was equal to CNY 74.8 thousand, the pollution reached the inflection point, and a decreasing trend appeared. Referring to the panel data, we found that the data of GDPPC higher than the turning point mainly appeared in the later periods of the time series, and the value added by industry as a percentage of GDP declined. The EKC result further proved that economic growth did not always increase PM_2.5_ concentrations in Liaoning Province, suggesting that changing economic growth mode was a correct choice for pollution control.

### 4.3. Implications for Regional Air Pollution Management

Through the study on the relationships between socioeconomic factors and PM_2.5_ concentration changes in Liaoning Province from 2000 to 2015, we found that the industrialization and economic growth were the main causes affecting the PM_2.5_ concentration changes from the perspective of short-term impacts and long-term contributions. As the traditional pillar industry of economic growth in Liaoning Province, the contributions of the secondary industry to regional pollution is predictable. According to the above data and results, we also found that the dependence of economic growth on the secondary industry in Liaoning Province was weakened, and the EKC curve also showed that economic growth did not always lead to the increase in PM_2.5_ concentrations. In 2014, the number of days of severe pollution (150–250 μg/m^3^) in Shenyang reached 22 days; in 2018, the number of days of severe pollution was only 2 days. Although there is still a big gap between China’s pollution level and the world standard, the improvement of atmospheric environment is obvious. This informs us that the transformation of economic structure is effective for the management of atmospheric pollution. However, improving energy efficiency and developing and utilizing clean energy is the key direction of taking into account both economic growth and environmental protection [49,50].

Among the socioeconomic variables, the urbanization process only showed the long-term impact on PM_2.5_ concentration changes, and the contribution was weak. In other words, the urban expansion and population growth had little direct effects on the changes of PM_2.5_ concentration, but indirectly affected the changes through economic growth and the industrialization process [19]. The causality diagram (Figure 5) showed that PM_2.5_ changes, industrialization and economic growth also affected the urbanization process in both the short and long term. As the level of urbanization in each period is closely related to the pollution exposure [51], the goal of “new-type urbanization” is not only to emphasize the rapid urbanization, but also to meet the health needs of residents [52]. Therefore, the study on relationships between regional environment and socioeconomic factors is necessary for the phased management of regional pollution, and more variables may be added according to the data availability and research objectives.

### 4.4. Limitations

The study results have explained the impacts of socioeconomic development on PM_2.5_ concentrations and the causal relationships among them to a large extent in Liaoning Province; however, there are still some limitations. For example, the surface PM_2.5_ data used in this study are the longest time series pollutant data available at present, but there is also a possibility that the lower spatial resolution of the data has affected the accuracy of the assessment results. If better data could be obtained (i.e., higher spatial resolution and longer time series), it would be beneficial to further explore the causes of regional and internal pollution differences in the future. On the other hand, complex coupling relationships among economic growth, urbanization, industrialization and PM_2.5_ concentrations were observed in this study. Determining how to decouple these relationships to further develop targeted solutions that tackle the pollution issue remains a challenging and urgent task. In the future, it is also necessary and meaningful to study and compare the relationships between policies and environment in other regions such as agriculture- or service-oriented areas and comprehensive areas.

## 5. Conclusions

In the panel data used in this study, the variables were all cointegrated. The Granger causality test results showed that economic growth, industrialization and urbanization were all long-term causalities of the changes of PM_2.5_ concentrations, and economic growth and industrialization also significantly affected changes in PM_2.5_ concentrations in the short term. The results of variance decomposition and the impulse response analysis showed that industrialization was the most important variable affecting PM_2.5_ concentrations. However, controlling only one socioeconomic factor to slow pollution growth is not feasible because there are either long-term or short-term and either bidirectional or unidirectional relationships among them. Though Liaoning Province has shown characteristics of economic and industrial transformation, it is also necessary to formulate more targeted policies to solve the problem of regional air pollution.

## Figures and Tables

**Table 1:** Description of the panel data from 2000 to 2015.

Variable	Obs.	Mean	Std. Dev	Min	Max
PM_2.5_ (μg/m^3^)	224	36.60	8.24	18.81	54.57
GDPPC (Yuan, RMB)	224	37,142.14	23,905.06	6184.72	121,457.46
UIS (%)	224	13.32	7.65	2.06	33.95
IND (%)	224	55.13	9.51	37.09	83.60

**Table 2:** Panel unit root test results.

Variable	Level		1st Difference	
	Intercept	Intercept and Trend	Intercept	Intercept and Trend
	Levin, Lin and Chu (LLC)			
*ln*PM_2.5_	−7.4320 ***	−2.6757 ***	−6.6609 ***	1.8893
*ln*UIS	−0.3350	−3.3226 ***	−6.8751 ***	−7.2374 ***
*ln*GDPPC	−13.618 ***	1.2149	−5.9671 ***	−17.066 ***
*ln*IND	1.4858	2.3637	−8.8109 ***	−6.2508 ***
	Im, Pesaran and Shin (IPS)			
*ln*PM_2.5_	−3.9769 ***	−0.5582	−5.9219 ***	−5.0734 ***
*ln*UIS	5.0142	−1.0338	−5.2980 ***	−4.2049 ***
*ln*GDPPC	−5.7427 ***	4.40875	−4.7635 ***	−11.892 ***
*ln*IND	2.1611	4.9270	−6.2508 ***	−6.3221 ***

Note: Significance: * 0.1, ** 0.05, *** 0.01.

**Table 3:** Panel cointegration test results using the Pedroni methods.

**Pedroni**	**Alternative Hypothesis: Common AR Coefs. (Within-Dimension)**				
		Statistic	Prob.	Weighted Statistic	Prob.
	Panel v-Statistic	1.2492	0.1058	1.0894	0.1380
	Panel rho-Statistic	0.0337	0.5134	−0.0132	0.4947
	Panel pp-Statistic	−1.9136 **	0.0278	−1.8940 **	0.0291
	Panel ADF-Statistic	−2.1804 **	0.0146	−2.5478 ***	0.0054
	**Alternative Hypothesis: Individual AR Coefs. (Between-Dimension)**				
		Statistic		Prob.	
	Group rho-Statistic	1.6771		0.9532	
	Group pp-Statistic	−1.7092 **		0.0437	
	Group ADF-Statistic	−3.0995 ***		0.0010	

Note: Significance: * 0.1, ** 0.05, *** 0.01.

**Table 4:** Panel fully modified least squares regression results.

Variable	Coefficient	Std. Error	t-Statistic
*ln*GDPPC	0.2620 ***	0.0025	104.2593
*ln*IND	0.2236 ***	0.0021	107.5758
*ln*UIS	0.0094 ***	0.0009	9.8713

R^2^ = 0.492128, Adj. R^2^ = 0.487221; Significance: * 0.1, ** 0.05, *** 0.01.

**Table 5:** Panel Granger causality test results.

Dependent Variable	Independent Variables					
	Short-Run Causality (χ2-Wald Statistics)				Long-Run Causality	
	Δ*ln*PM_2.5_	Δ*ln*GDPPC	Δ*ln*UIS	Δ*ln*IND	ECT (−1)	*t-statistics*
Δ*ln*PM_2.5_		6.2655 **	1.7088	5.2909 *	−0.0665 ***	−5.6409
Δ*ln*GDPPC	12.0662 ***		0.9156	2.3951	−0.0704 ***	−3.2335
Δ*ln*UIS	6.1390 **	14.3349 ***		9.3067 ***	−0.0272 **	−2.3615
Δ*ln*IND	2.6420	14.4685 ***	1.0221		0.0072 ***	2.8231

Significance: * 0.1, ** 0.05, *** 0.01.

**Table 6:** Variance decomposition analysis results of pm_2.5_ concentrations in the panel.

Period	S.E.	*ln*PM_2.5_	*ln*GDPPC	*ln*IND	*ln*UIS
Variance Decomposition of *ln*PM_2.5_:					
1	0.068920	100.0000	0.000000	0.000000	0.000000
2	0.097998	98.17410	1.265334	0.488164	0.072398
3	0.114122	97.05163	2.419547	0.366743	0.162078
4	0.123131	95.67872	3.687611	0.410396	0.223269
5	0.128520	94.08125	4.921052	0.746677	0.251024
6	0.132068	92.35911	6.035212	1.349410	0.256273
7	0.134648	90.63484	6.970944	2.143034	0.251183
8	0.136672	89.00029	7.711078	3.044643	0.243994
9	0.138337	87.50652	8.266401	3.988203	0.238875
10	0.139744	86.17206	8.662361	4.928241	0.237340
11	0.140948	84.99492	8.928941	5.836583	0.239554
12	0.141987	83.96262	9.095029	6.697198	0.245149
13	0.142889	83.05865	9.185900	7.501821	0.253625
14	0.143674	82.26610	9.222504	8.246885	0.264513
15	0.144360	81.56939	9.221616	8.931577	0.277415
16	0.144962	80.95495	9.196351	9.556689	0.292006
